# Hypnotherapy for agoraphobia—Feasibility and efficacy investigated in a pilot study

**DOI:** 10.3389/fpsyg.2023.1213792

**Published:** 2023-08-10

**Authors:** Kristina Fuhr, Annika Bender, Ariane Wiegand, Paul Janouch, Marta Drujan, Barbara Cyrny, Cornelie Schweizer, Benjamin Kreifelts, Vanessa Nieratschker, Anil Batra

**Affiliations:** ^1^Department of Psychiatry and Psychotherapy, Tübingen Center for Mental Health, University Hospital of Tübingen, Tübingen, Germany; ^2^Max Planck Fellow Group Precision Psychiatry, Max Planck Institute of Psychiatry, Munich, Germany; ^3^Outpatient Psychotherapy Practice, Bad Salzuflen, Germany; ^4^German Center for Mental Health (Deutsches Zentrum für Psychische Gesundheit), University Hospital of Tübingen, Tübingen, Germany

**Keywords:** agoraphobia, psychotherapy, hypnotherapy, (epi-)genetic, feasibility

## Abstract

A number of case studies describing hypnotherapy in the treatment of anxiety disorder patients have already been published. Only a few randomized controlled trials (RCTs) investigated the efficacy of hypnotherapy but focused mainly on symptoms rather than specific mental disorders. The goal of this study was to investigate whether hypnotherapy (HT) was superior to a waitlist control group (WL) in the reduction of agoraphobia-related symptoms. Further goals were to report the feasibility of hypnotherapy as well as attrition and completion rates and detect (epi-)genetic variables, which might play a role in treatment outcome. This pilot study was based on a monocentric two-armed randomized controlled rater-blind clinical trial that was conducted between 2018 and 2020 with a waitlist control group. A total of 36 patients diagnosed with agoraphobia were randomized to either HT or WL. Patients in HT received individual outpatient treatment with hypnotherapy with 8 to 12 sessions for a period of 3 months. Patients in WL received HT after 3 months. Agoraphobia-related symptoms were assessed at baseline, after the treatment, and 3 months later in both groups with a clinician rating. The primary hypothesis concerning the difference between groups in the individual percentage symptom reduction could be confirmed in the intention-to-treat, not the per-protocol sample. Additionally, we applied repeated-measures analyses of variance and found a higher symptom decrease in HT compared with WL patients in three of the five imputed datasets. The dropout rate was low, and satisfaction with the treatment was high. HT patients experienced a strong symptom reduction after receiving hypnotherapy. WL patients improved slightly during the waiting period. The *COMT* Val^108/158^Met genotype had an effect on the agoraphobia-related symptoms as well as on *COMT* DNA methylation levels. This is the first study to indicate that hypnotherapy performed better than a waitlist control group regarding the reduction in anxiety symptoms in an RCT. Future studies should confirm the efficacy of hypnotherapy and compare the treatment with a standard treatment for anxiety disorders in a larger trial. Future studies should also investigate whether hypnotic susceptibility is associated with *COMT* Val^108/158^Met genotype and could predict treatment success for HT.

**Clinical trial registration:**

https://classic.clinicaltrials.gov/ct2/show/NCT03684577, identifier: NCT03684577.

## 1. Introduction

Mental disorders in Europe are estimated to affect ~164.8 million citizens, which corresponds to more than one-third of the population per year (Wittchen et al., 2011). For agoraphobia with and without panic disorders, lifetime prevalence rates of 2.6% were found in adults with higher rates in women compared to men in the United States (Kessler et al., 2012). In Europe, about 2% are diagnosed with agoraphobia (Goodwin et al., 2005; Wittchen et al., 2011). Concerning Germany, the 12-month prevalence for anxiety disorders is almost 16 and 4% for agoraphobia, with women showing higher prevalence rates than men (Jacobi et al., 2014). The current national and international guidelines regarding the treatment of anxiety disorders (National Institute for Health Care Excellence, 2011; Bandelow et al., 2014, last updated 2020) recommend psychotherapy alternatively to pharmacological treatment with the highest evidence. The psychotherapy with the highest evidence is CBT (National Institute for Health Care Excellence, 2011; Bandelow et al., 2014). Most efficacious treatments, such as CBT, include exposure techniques (see Kaczkurkin and Foa, 2015), and CBT with exposition *in vivo* therefore is considered to be the gold standard treatment in agoraphobia (National Institute for Health Care Excellence, 2011; Kaczkurkin and Foa, 2015). Despite the high evidence for CBT, response rates across several studies in anxiety disorders are only ~50% (Loerinc et al., 2015). Up to one-third of patients with panic disorder and agoraphobia report residual symptoms 2 years after treatment with CBT (Gloster et al., 2013). To improve outcomes, alternative or new treatments have been developed, and compared with CBT, for example, interpersonal psychotherapy (IPT), new treatment strategies added to CBT, or comparing traditional forms (face to face) with internet-based delivered psychotherapy. In one comparison, CBT was superior to IPT in patients with panic disorder with agoraphobia concerning the primary outcome, such as the frequency of panic attacks, but not the secondary outcomes such as anxiety cognitions and feelings (Vos et al., 2012). Internet-based CBT was as effective as CBT delivered face to face in a pilot study (Kiropoulos et al., 2008). Comparing the “traditional” form of psychotherapy with CBT, short-term psychodynamic psychotherapy of panic disorder with and without agoraphobia seems to be comparably effective as CBT in a comparative review, but the included studies showed high risk of bias (Papola et al., 2022). Recently, interventions with imagery rescripting have been evaluated regarding their efficacy for anxiety disorders, as, for example, social phobia or PTSD (Arntz, 2012; Strachan et al., 2020), but not (yet) agoraphobia.

Hypnotherapy could offer an alternative treatment option. Concerning the treatment of agoraphobia, some advantages of hypnotherapy can be identified. Techniques, such as the imagination of an inner safe place, showed good results in the treatment of post-traumatic stress disorders (PTSD, Zehetmair et al., 2018). The safe place is also recommended as a strategy in schema therapy for personality disorders (Arntz, 2011) and is commonly used at the beginning of treatment with hypnotherapy. In hypnotherapy, trance inductions could be used to strengthen experiences in an exposure-based treatment in sensu, as, for example, recommended by Wolpe (1964) for introducing systematic desensitization. Thus, the imagined experience can be amplified and thereby modify perception and somatic responses, as outlined by Spiegel (2013). As another advantage, with hypnotic dissociation, the psychological and physiological aspects of anxiety can be compartmentalized (2013). If the situation triggering the initial agoraphobia is not consciously known, the use of hypnotic regression may be helpful. Comparable to imagery rescripting (Arntz, 2012), a past negative experience can be replaced by a new desired course of the event using hypnotic regression and reparenting. A new hypnotherapy approach included both stabilization techniques and also hypnotic regression similar to imagery rescripting and was initially published as a chapter in a German book on hypnosis (Revenstorf and Peter, 2015, Chapter 35). However, evidence for hypnotherapy in the treatment of specific anxiety disorders is scarce. In the only RCT with the primary diagnosis of agoraphobia and panic disorder, a standard exposure treatment was compared with an additional self-hypnosis training in a crossover design (Van Dyck and Spinhoven, 1997). The combined treatment, however, did not show superiority to the exposure treatment (Van Dyck and Spinhoven, 1997). Imagery rescripting, hypnotic regression, or inducing a safe place, however, were not part of their hypnosis training. In a recent study (Calzeroni and Giacosa, 2019), hypnotherapy was compared with cognitive therapy in the treatment of panic disorder. There were no differences between both treatments in clinical outcomes. However, the allocation was not random, which limits the interpretability of the results. Even though there are treatment concepts that add hypnotherapy methods, such as hypnotic trance, posthypnotic suggestions, and imagery, to CBT treatments, such as desensitization and exposure (Golden, 2007, 2012; Alladin, 2016), there are no RCTs to show empirical support. For example, Golden (2007, 2012) introduced a combined cognitive therapy with techniques of hypnotherapy but also criticized that evidence-based trials are missing. The meta-analysis by Ramondo et al. (2021) updated the results for hypnosis as an adjunct treatment to CBT and also included results from unpublished doctoral dissertations. However, none of the studies included in this meta-analysis treated patients with agoraphobia, and overall, the effect size for the CBTH combination was not superior compared to CBT alone in the treatment of “anxiety” (including phobias such as public speaking and test anxiety, dental anxiety, stress disorders, and other non-clinical samples) (Ramondo et al., 2021). A meta-analysis by Valentine et al. (2019) concluded that hypnotherapy can reduce symptoms of anxiety. However, no study included patients with a clinically confirmed diagnosis of an anxiety disorder according to international classifications and the allocation to the treatment in most of the studies was not random. Case reports show the first indications of the feasibility and acceptance of hypnosis (Gruenewald, 1971; Harris, 1991; Kraft, 2011). Some of those describe the treatment with hypnotherapy in agoraphobia and panic disorders and also used hypnotic regression (Gruenewald, 1971; Delmonte, 1995). Indirect evidence for the effects of hypnosis in reducing anxiety could be found by reduced activation of the related brain areas of the fear network (anterior cingulate cortex, insula, and also the hippocampus) during hypnosis in patients with dentist phobia (Halsband and Wolf, 2015). When providing safety during hypnosis, high suggestible participants showed a reduced response to rewards as measured by a reduction of the amplitude of a P300 in a risk task (Schmidt et al., 2020). Up to date, no clear evidence-based implications can be drawn.

The etiology of anxiety disorders is influenced by genetic as well as environmental factors, e.g., stressful live events (Hettema et al., 2001; Faravelli et al., 2012) and interactions between them (G × E, Nugent et al., 2011). One mediator of those G x E interactions is the epigenetic regulation of gene expression (Bartlett et al., 2017). The best studied epigenetic mechanism is DNA methylation (DNAm), the covalent modification of cytosine in a cytosine–guanine dimer (CpG site). DNAm of a promoter region is generally associated with decreased expression of the concerned gene (Jones, 2012).

Genetic variance and differential DNA methylation in several genes have been reported as being associated with agoraphobia and panic disorder (e.g., Lueken et al., 2016; Gottschalk and Domschke, 2017; Schiele et al., 2020). An interesting variant in this context is the *COMT* Val^108/158^Met polymorphism, which has been associated with anxiety susceptibility and anxiety-related traits (Stein et al., 2005; Baumann et al., 2013; Howe et al., 2016). Furthermore, it has also been found associated with hypnotizability; however, the results are partly contrary regarding the direction of the effect (Lichtenberg et al., 2000, 2004; Szekely et al., 2010; Rominger et al., 2014; Storozheva et al., 2018). Still, this remains an intriguing discovery with regard to the potential option of personalized psychotherapy. There is increasing evidence that epigenetic markers could also prove to be useful in the context of personalized psychotherapy, as some studies reported epigenetic effects correlating with psychotherapeutic treatment success in anxiety disorder patients (Eley et al., 2012; Roberts et al., 2014, 2015, 2019; Ziegler et al., 2016, 2019; Moser et al., 2022). However, for hypnotherapy, no such (epi-) genetic approaches have been reported yet.

The purpose of this study was to examine, in a randomized controlled trial of patients with the diagnosis of agoraphobia according to DSM-5, if hypnotherapy (HT) results in a higher symptom reduction in anxiety in a clinician-rating compared to a waitlist control group (WL). We will also report results concerning the WL after they received the HT treatment as well as the 3-month follow-up for patients initially receiving HT. Furthermore, we examined feasibility, attrition and completion rates, and safety. In a subsample of the patients, the potential of the *COMT* gene to function as an (epi-)genetic marker for hypnotherapeutic success was evaluated. Participant's *COMT* Val^108/158^Met genotype and changes in *COMT* DNA methylation (DNAm) over the course of the intervention were assessed to investigate the predictive value of genetic and/or epigenetic factors on response to hypnotherapy.

## 2. Methods

### 2.1. Trial design

The clinical pilot study was based on a 2 × 2 mixed design with the factor time (pre and post) and the factor treatment condition (HT vs. WL). Additionally, the assessments were repeated 3 months after post (3 months follow-up for HT, respectively, postassessment for WL). A blockwise randomization sequence was created using nQuery 7.0 (Statsols, Cork, Ireland) for up to 50 patients by an external institute for biometry and clinical epidemiology. The authors assert that all procedures contributing to this study comply with the ethical standards of the relevant national and institutional committees on human experimentation, with the Helsinki Declaration of 1975, as revised in 2008, and with the General Data Protection Regulation of the European Union. All procedures involving human subjects/patients were approved by the Ethics Committee of the University Hospital Tübingen (546/2018BO2). The trial was registered with ClinicalTrials.gov before recruiting participants (NCT03684577).

### 2.2. Trial sample

**Figure 1:**
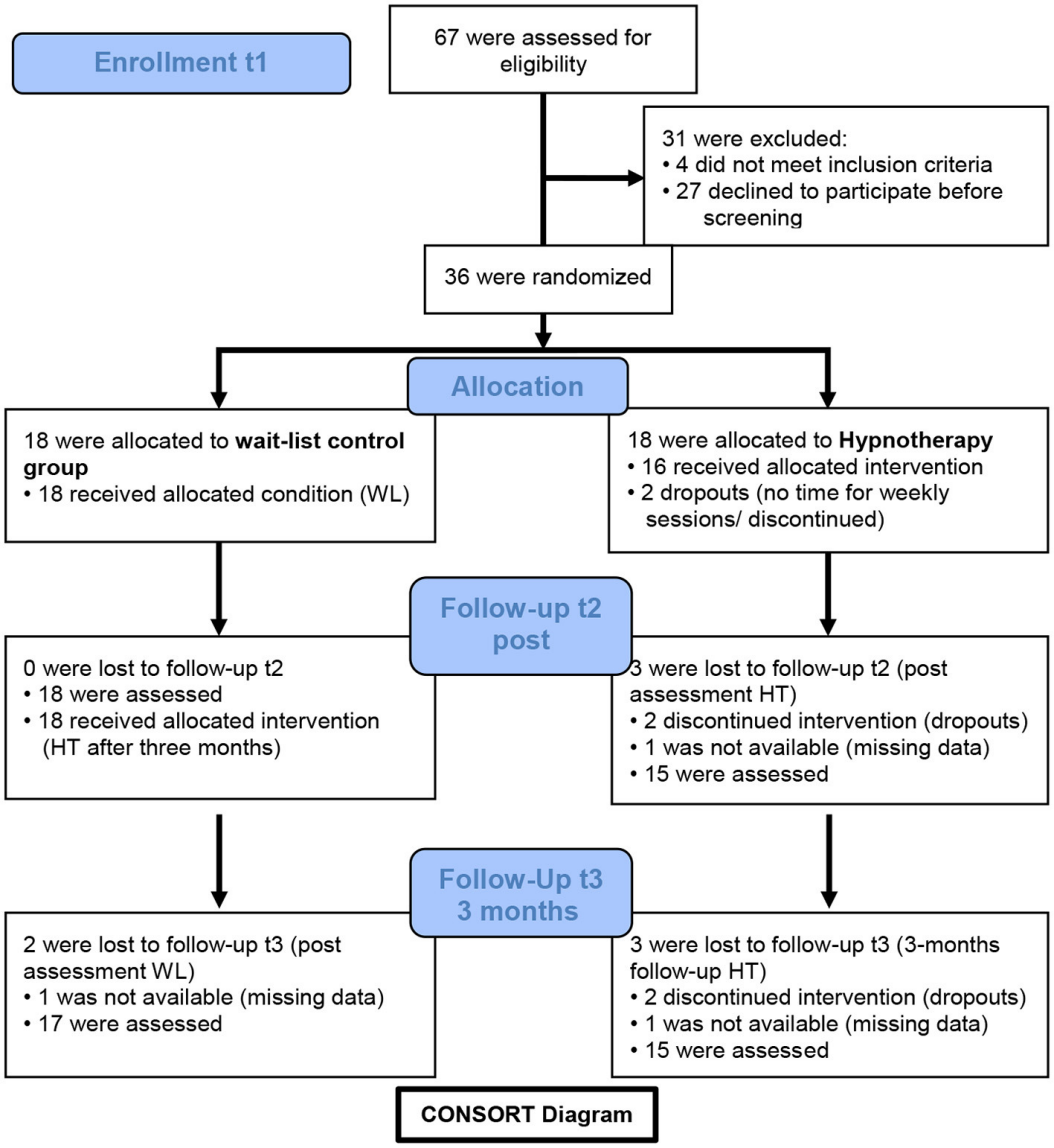
CONSORT diagram. Trial enrollment, randomization, and follow-ups.

During the recruitment period, a total of four e-mails announcing the study were successively sent to all members of the university and the university hospital (probably over 30,000 recipients altogether), two announcements were placed in the local newspapers, and flyers and posters were sent and distributed to pharmacies and hospitals around Tübingen. The main inclusion criterion was the diagnosis of current agoraphobia according to the Diagnostic and Statistical Manual of Mental Disorders—Fifth Edition (DSM-5, American Psychiatric Association, 2013). A further inclusion criterion was being at the age of 18–65 years (the official age for employment in Germany at that time). We excluded patients with a lifetime diagnosis of a bipolar disorder or psychotic disorder, acute suicidality (intended action, concrete plans, or intermittent pronounced suicidal ideation), drug or alcohol use disorder in the last 12 months, or if patients had other severe primary mental disorders (for example, the diagnosis of a current major depressive episode, personality disorder of borderline type with self-injury, actual post-traumatic stress disorder, or anorexia nervosa), if patients were on anxiolytic medication, and if patients attended another outpatient psychotherapy during the last 12 months. The Mini-International Neuropsychiatric Interview (M.I.N.I., Sheehan et al., 1998), adapted for DSM-5, was used to assure inclusion and exclusion criteria for the study and to assess potential comorbid psychiatric disorders. Comorbid disorders, like panic attacks, panic disorder, social anxiety, major depression lifetime, or an obsessive-compulsive, dependent, or insecure personality disorder, were allowed. An antidepressant medication that is also approved for the treatment of anxiety disorders was allowed in case medication had been stable for at least 8 weeks prior to study inclusion. In total, 67 patients were interested in participation. Of these, 27 patients declined participation before screening. Reasons were that patients were currently in psychotherapy treatment (*n* = 5), had another anxiety problem (*n* = 8, such as fear of spiders, dogs, heights, obsessive–compulsive disorder, or social anxiety), refused to participate (*n* = 4), or there were other reasons (*n* =10) why patients could not attend the screening. A total of 40 patients were screened for eligibility. After screening, four patients were excluded because they did not meet inclusion criteria or refused to participate after screening. In total, 36 patients were included in the trial and randomized to either HT (*n* = 18) or WL (*n* = 18). For details on the patient flow, see the CONSORT diagram in Figure 1.

### 2.3. Assessments

#### 2.3.1. Primary endpoint

The primary endpoint of the study was the 13-item clinician-rating Panic and Agoraphobia Scale (PAS, Bandelow, 1999), assessed at baseline (t1) and the end of the treatment (t2) comparing HT and WL. The primary outcome was the percentage symptom reduction in the PAS score calculated for each patient separately. The internal consistency at baseline was high with α = 0.85. The inter-rater reliability for the PAS score between two raters was ICC = 0.97 with a randomly selected sample of n = 27 interviews of the three different raters (nine PAS interviews of each rater) who were involved in the trial.

#### 2.3.2. Secondary endpoints

##### 2.3.2.1. Attrition and completion rates

Dropout was defined as withdrawal from participation after randomization, discontinuation of the study treatment before eight sessions, and missing data at the assessment after the end of the intervention.

##### 2.3.2.2. Satisfaction

Satisfaction at the end of the treatment regarding the treatment and the therapist was assessed with visual analog scales (range 0–100, numerically transformed). To ensure the blindness of raters, patients were encouraged to complete the satisfaction rating individually after their treatment (HT patients after 3 months; WL patients after 6 months) and send it back to the study center in a stamped envelope.

##### 2.3.2.3. Safety

During the trial, serious adverse events (SAEs) were assessed. SAEs were defined as (1) a lethal event, (2) suicidal ideations, (3) hospitalization for somatic reasons for 24 h or more, (4) hospitalization for psychiatric reasons, (5) invalidity, and (6) any other medically relevant state.

All assessments were conducted at baseline (t1), postassessments 3 months later (t2), and the follow-up after 3 months (t3). For epigenetic assessments, another follow-up for only the WL group 3 months after receiving HT (t4) was conducted.

##### 2.3.2.4. (Epi-)genetic methods/assessments

All randomized patients in the study were invited to take part in the (epi-)genetic assessments, which was described as an additional voluntary study. Participants of the (epi-)genetic pilot study received 10 € to compensate for their time.

DNA of the participants was obtained using Oragene OG-500 saliva collection tubes (DNA Genotek™; Ottawa, Canada). The DNA isolation and purification were performed using a prepIT.L2P kit (DNA Genotek™) according to the manufacturer's protocol. DNA quantity was measured using Qubit^®^ dsDNA BR Assay Kit (Thermo Fisher Scientific Inc.; Waltham, USA).

Sodium bisulfite conversion for epigenetic analysis was performed using EpiTect^®^ Fast DNA Bisulfite Kit (QIAGEN GmbH; Hilden, Germany) according to the manufacturer's protocol.

Amplification of the targeted sequence was performed by PCR using PyroMark PCR Kit (QIAGEN GmbH) following the manufacturer's instructions. The primer design was adopted by Mill et al. (2006), with the reverse primer containing a biotin tag at its 5′ end. To test for successful amplification, the PCR products were run on a 2% agarose gel.

Analysis of the DNAm at the two CpG sites of interest (GRCh38/hg38 chr22: 19,962,527–19,962,567) was performed by pyrosequencing using the PyroMark Q24 system (QIAGEN GmbH, software version 2.0.7) according to the manufacturer's protocol. Two technical replications of DNAm levels per analyzed CpG site, differing by no more than 3%, were assessed per time point and participant.

Identification of the participant's *COMT* Val^108/158^Met genotypes was performed as described in Thomas et al. (2019). Accuracy was assessed by duplicating 15% of the original sample, and reproducibility was 100%. The genotype frequencies did not deviate from Hardy–Weinberg equilibrium (HWE; *p* = 0.12).

### 2.4. Hypnotherapy

Hypnotherapy consisted of 8–12 individual sessions of 50 min each over a period of 3 months. Up to three double sessions were allowed to compensate for breaks or to intensify the hypnotic experience. HT for agoraphobia is based on the theoretical humanistic assumption that agoraphobia-related symptoms arose as a positive solution strategy to overcome a personal problem in the lifetime history. Thus, the most important module of HT is based on a hypnotic symptom regression technique to reframe the past problematic situation in a more constructive way similar to imagery rescripting. For example, after exploring the past situation, patients were offered to either solve the last situation (writing an imagery script for a new end/continuing the frozen script), or realize that they overcame and survived this situation, or negotiate questions of guilt. Other modules of HT include the introduction of a safe place, hypnotic activation and reinforcement of personal resources, and the use of relevant positive experiences from the biography as stabilization techniques at the beginning of the treatment. HT was embedded in a cognitive-behavioral (CBT) framework and also included psychoeducation about hypnosis and agoraphobia using a psychophysiological model to explain anxiety symptoms, similar to CBT. Other techniques were formal trance induction, utilization techniques, metaphors, and posthypnotic suggestions. Ideomotor signals, such as an arm or hand levitation, were used to indicate non-verbal responses and to intensify the hypnotic experience. Other CBT techniques, such as systematic desensitization, *in vivo* exposure, or addressing and modifying maladaptive thoughts were not part of the HT treatment.

Two female therapists with a certificate in clinical hypnosis and with more than 10 years of professional experience with hypnotherapy received intensive training in the treatment manual. The therapists were 50 and 55 years old. The therapists were responsible for the treatment of all patients (including the 18 patients of WL after their 3-month waiting period).

Treatment fidelity was assessed at the end of the trial by two raters who were not involved in the treatment at any time. The raters were trained in the treatment manual and the fidelity ratings in a 1-day training. They listened to 48 randomly selected therapy sessions (BK: *n* = 26, SP: *n* = 22). Inter-rater reliability was calculated across eight randomly selected sessions that were rated by both raters and was very high with ICC = 0.92. HT fidelity consisted of up to 11 different techniques that could be applied in any HT session (resource activation, formal trance induction, seeding with meaningful messages, use of metaphors, work with time progression/regression, posthypnotic suggestions, externalization, utilization, ideomotor signs, association/dissociation, and psychoeducation). The items for the HT fidelity were developed during another trial regarding the treatment of major depression with HT (Fuhr et al., 2021). The 11 different techniques could be rated with a frequency of 1–3 for each treatment session resulting in an overall score of a maximal 33. In total 60% of the rated sessions had a score of 15 or higher with an average M = 16.10 (SD = 7.84). On average, six of eleven techniques were used in the treatment sessions (M = 5.88, SD = 2.73, Median = 6.00). Raters [*t*_(46)_ = 0.06, *p* = 0.950] and therapists [*t*_(46)_ = −1.05, *p* = 0.301] did not differ in the ratings of fidelity.

### 2.5. Procedure

Patients were recruited and screened between October 2018 and January 2020 at the study site at the University Hospital of Psychiatry and Psychotherapy, Tübingen. Written informed consent was obtained from all the patients after the procedures for participating in the trial had been fully explained. Afterward, eligible participants were randomly assigned 1:1 to either HT or WL. Patients were also invited to take part in the (epi-)genetic study before their first session.

Treatment assignment for each patient was communicated via email between the statistical center of the trial (IKEAB) and the study center shortly after inclusion. The details of the randomization sequence were unknown to the investigator, the coordinator, and the therapists. Follow-up assessments took place after 3 months (postassessment for HT patients, t2) and another 3 months later (follow-up for the HT patients, postassessment for the WL patients after receiving the treatment, t3). For an overview of the timeline, see the CONSORT, Figure 1. Raters at follow-ups were blind concerning the treatment condition of the patient. A total of three raters were involved in the baseline and two in the follow-up assessments. Raters had at least a bachelor's degree in psychology, participated in a course in clinical interviewing at university or elsewhere, and underwent specific half-day training in the interviews for this study. With patient consent, baseline and follow-up interviews as well as therapy sessions were recorded on digital audio-tapes to calculate the inter-rater-reliability of the PAS between the original rating and a blind second rating and for the assessment of treatment fidelity. The raters documented whether they were unblinded by the patients at follow-ups. The last therapy sessions and ratings for the post-test in HT were conducted in March and April 2020, respectively, in July 2020 in WL, with the last four patients switching to video therapy or telephone-based clinical interviews because of the COVID-19 lockdown in Germany. The investigators and authors of the study were blinded with respect to the results until the database was closed in September 2020.

Saliva sampling was conducted at the University Hospital of Psychiatry and Psychotherapy, Tübingen, for t1. At post and follow-up, sampling sets were mailed to the participants, who collected their saliva independently, before sending the sampling back to the laboratory.

### 2.6. Statistical analysis plan

#### 2.6.1. Power analysis

Assumptions were made for a one-tailed *t*-test between two independent groups with an expected large effect size (*d* = 0.80) based on the results summarized in Bandelow et al. (2014, p. 35), an alpha level of α =0.05 and a power (1 – β) of 86%. With a 1:1 allocation, a sample size of 24 patients in each of the two groups (total *N* = 48) would have a current power of 86% (non-centrality parameter δ = 2.77, critical *t* = 1.68). Sample size calculation was conducted using G^*^Power (Faul et al., 2007).

#### 2.6.2. Analysis of the primary endpoint

The primary analysis should be based on the primary endpoint, the individual percentage improvement in the PAS score, conducted with the intention-to-treat (ITT) sample with all patients being randomized in the trial. Since the normal distribution of the individual percentage improvement was violated, we decided to use non-parametric Mann–Whitney U-tests instead of the initially planned one-sided independent *t*-tests. *P*-values will be reported one-sided (divided by 2). For the ITT analysis, we decided to replace missing data with the multiple imputations method (MI). Thus, after assuring that the missing data of the primary outcome measure were random, we generated five imputed datasets based on a linear regression imputation algorithm automatically generated by SPSS. The primary analyses were conducted separately for each of the five imputed data sets. The descriptive values of the five imputations will be reported separately. Analysis of the per-protocol (PP) sample served as a sensitivity analysis. Treatment participation was considered as PP if the patient attended eight or more sessions, and complete data were available at the postassessment after 3 months (t2). Single missing values in PAS items (*n* = 4 at t1, 3 at t2, and 2 at t3) were replaced by regression estimates.

#### 2.6.3. Analysis of secondary endpoints

As power was low for our primary analysis, we conducted a repeated-measures analysis of variance (rmANOVA) regarding the PAS pre and post scores between both groups which were normally distributed, reporting two-sided significance results and aggregated descriptive values of the imputed datasets. We also compared the improvement in the PAS pre–post and differences between groups at post with Cohen's *d* effect sizes. As another exploratory analysis, we compared the symptoms between groups at the 3 months follow-up t3 (respectively, postassessment for the WL patients).

We will report the satisfaction with the treatment as well as completion and attrition rates with reasons for discontinuation as well as the number and type of reported SAEs.

The statistical analysis was performed with SPSS^®^ Statistics 27.0 (IBM^®^ Ehningen, Germany). The authors and investigators of the current trial were blind concerning the primary endpoint until the database was closed in August 2020.

#### 2.6.4. (Epi-)genetic analyses

For epigenetic analyses, the participants from HT and WL were combined to compare *COMT* DNAm levels at pre, post, and follow-up (t1–t3 for HT; t2–t4 for WL). Statistical data analysis was also performed using SPSS^®^ Statistics 27.0 (IBM^®^ Ehningen, Germany). The technical replications of DNAm were averaged for statistical analyses. After confirming the correlation of the DNAm at both assessed CpG sites, the means were averaged to calculate an overall DNAm mean of the *COMT* promoter region per time point and participant, in the following, referred to as DNAm at pre, post, and follow-up, respectively. Three variables deviated from normal distribution according to the Shapiro–Wilk test (“PAS score at t3,” “mean DNAm at t1,” and “mean DNAm at t3”). Thus, to conduct a non-parametric alternative of a rmANOVA, including interaction terms, van der Waerden's normal scores of the ranks of the dependent variable used in the respective model were utilized, as has been proposed before (Conover and Iman, 1981; Zimmerman and Zumbo, 1993; Mansouri and Chang, 1995). To ensure comparability of the results, this approach, in the following referred to as NSrmANOVA, was applied for all comparisons of PAS and DNAm, including normally distributed dependent variables. After rank transformation, normality was confirmed, again by Shapiro–Wilk test. Sphericity was tested using Mauchly's test. In the case of a significant Mauchly's test, Greenhouse–Geisser correction was applied for Greenhouse–Geisser ε < 0.75 (Girden, 1992).

## 3. Results

### 3.1. Feasibility, attrition, and completion rates

The dropout rate in HT was low (*n* = 3, 16.67%). One HT patient did not attend any therapy session even if different appointments were proposed by the therapist. Another HT patient developed some trauma-related new symptoms (see also the details of the SAEs), which led to hospitalization and treatment dropout after three sessions with the therapist. The third HT patient completed the treatment successfully but was not available for the postassessment at t2. All patients of WL completed the assessment at t2. Therefore, the PP sample at t2 was *n* = 33 (HT: *n* = 15, WL *n* = 18).

At the follow-up t3 (postassessment for WL patients after receiving HT), the two HT treatment dropouts were not assessed as well as another HT patient. In WL, two patients were not available for the assessment. The number of completed data at t3 was *n* = 31 (HT: *n* = 15, WL *n* = 16).

All HT patients attended on average M = 11.25 (SD = 1.00, *n* = 16) sessions with a range of 9–12 as intended according to the documentation of the therapists. All WL patients received the HT treatment after the waiting period and attended M = 11.00 (SD = 1.57, *n* = 18, range 7–12) sessions.

### 3.2. Sample characteristics

The characteristics of the ITT sample are displayed in Table 1. Patients were on average 42.03 (SD = 15.14) years old. Six participants were on antidepressant medication during the trial. None had an anxiolytic medication. About half of the patients showed current comorbid anxiety disorders, mostly panic attacks or panic disorder, for details also on the characteristics of the epigenetic subsample (see Table 1). The participants of the (epi-)genetic subsample (*n* = 17, 14 women and 3 men) were on average 36.65 (SD = 14.17) years old. Of those, ten were HT patients and seven were WL patients receiving HT after 3 months.

**Table 1:** Characteristics of the trial sample (ITT, *n* = 36).

**Variables**	**HT (*n* = 18)**	**WL (*n* = 18)**	**Epigenetic sample (*n* = 17)**	**Total (*n* = 36)**
	**M (SD)**	**M (SD)**	**M (SD)**	**M (SD)**
Age	40.28 (13.33)	43.78 (16.97)	36.65 (14.17)	42.03 (15.14)
	No. (%)	No. (%)	No. (%)	No. (%)
Sex, female	17 (94.44)	12 (66.67)	14 (82.35)	29 (80.56)
Antidepressant medication (AD)	3 (16.67)	3 (16.67)	3 (17.65)	6 (16.67)
SSRI	2 (11.11)	3 (16.67)	3 (17.65)	5 (13.89)
Tricyclic AD	1 (5.56)	0 (0.00)	0 (0.00)	1 (2.78)
Comorbidity	16 (88.89)	14 (77.78)	8 (47.06)	30 (83.33)
Current^a^	11 (61.11)	8 (44.44)	5 (29.41)	19 (52.78)
Panic disorder	9 (50.00)	3 (16.67)	3 (17.65)	12 (33.33)
Panic attacks	2 (11.11)	3 (16.67)	0 (0.00)	5 (13.89)
Social phobia	0 (0.00)	1 (5.56)	0 (0.00)	1 (2.78)
Obsessive PS	0 (0.00)	1 (5.56)	0 (0.00)	1 (2.78)
Only lifetime/previous^a^	7 (38.89)	11 (61.11)	11 (64.71)	18 (50.00)
Panic disorder	3 (16.67)	6 (33.33)	5 (29.41)	9 (25.00)
Panic attacks	0 (0.00)	1 (5.56)	1 (5.88)	1 (2.78)
Major depression	4 (22.22)	4 (22.22)	5 (29.41)	8 (22.22)

M, mean; SD, standard deviation; No., number; HT, hypnotherapy; WL, waitlist control group; SSRI, selective serotonin reuptake inhibitor; PS, personality disorder.

a Double entry possible meaning a patient could have a current but also a lifetime/previous comorbid disorder.

At t2, five patients in HT and six in WL revealed their treatment condition to the rater (*n* = 11, 33.3%). However, the fact that raters were unblinded had no effect on the primary outcome, *r* = 0.05, *p* = 0.775 and was equally distributed between HT and WL, χ^2^ (1) = 0.01, *p* = 0.998.

### 3.3. Primary outcome

For the means and standard deviations of the PAS scores in both groups at all three assessments, as well as the primary outcome of individual percentage symptom reduction (medians are displayed) (see Table 2).

**Table 2:** Primary and secondary outcomes in the PP and ITT samples.

**Variables**	**PP sample (*****n*** = **33)**		
	**HT (*n* = 15)**	**WL (*n* = 18)**	**Total (*n* = 33)**
	**M (SD)**	**M (SD)**	**M (SD)**
PAS pre (t1)	13.11 (9.05)	11.69 (8.22)	12.33 (8.50)
PAS post (t2)	7.43 (6.16)	10.87 (6.13)	9.31 (6.29)
PAS follow-up (t3)^a^	6.87 (6.83)	7.38 (7.57)^a^	7.13 (7.11)^b^
16.5-1,15.5498pt	**Md (Range)**	**Md (Range)**	**Md (Range)**
PAS percentage improvement t1–t2	33.33 (400.00)	6.90 (307.72)	18.75 (400.00)
	**HT (*****n*** = **18)** **M (SD)**	**ITT sample (*****n*** = **36, MI)** **WL (*****n*** = **18)** **M (SD)**			**Total (*****n*** = **36)** **M (SD)**
PAS pre (t1)	14.46 (9.49)	11.69 (8.22)	13.08 (8.86)
PAS post (t2)	7.88 (6.19)	10.87 (6.13)	9.38 (6.28)
PAS follow-up (t3)	6.87 (6.80)	7.52 (7.30)	7.20 (7.05)
	**Md (Range)**	**Md (Range)**	**Md (Range)**
PAS percentage improvement t1–t2	33.05–36.05 (400–424.47)	6.90 (307.72)	19.38–23.89 (400–424.47)

M, mean; SD, standard deviation; Md, median; PAS, Panic and Agoraphobia Scale; HT, hypnotherapy; WL, waitlist control group; PP, per protocol; ITT, intention to treat; MI, multiple imputation (five datasets). PAS scores for the ITT sample were aggregated (on average).

a WL with *n* = 16.

b With *n* = 31.

**Figure 2:**
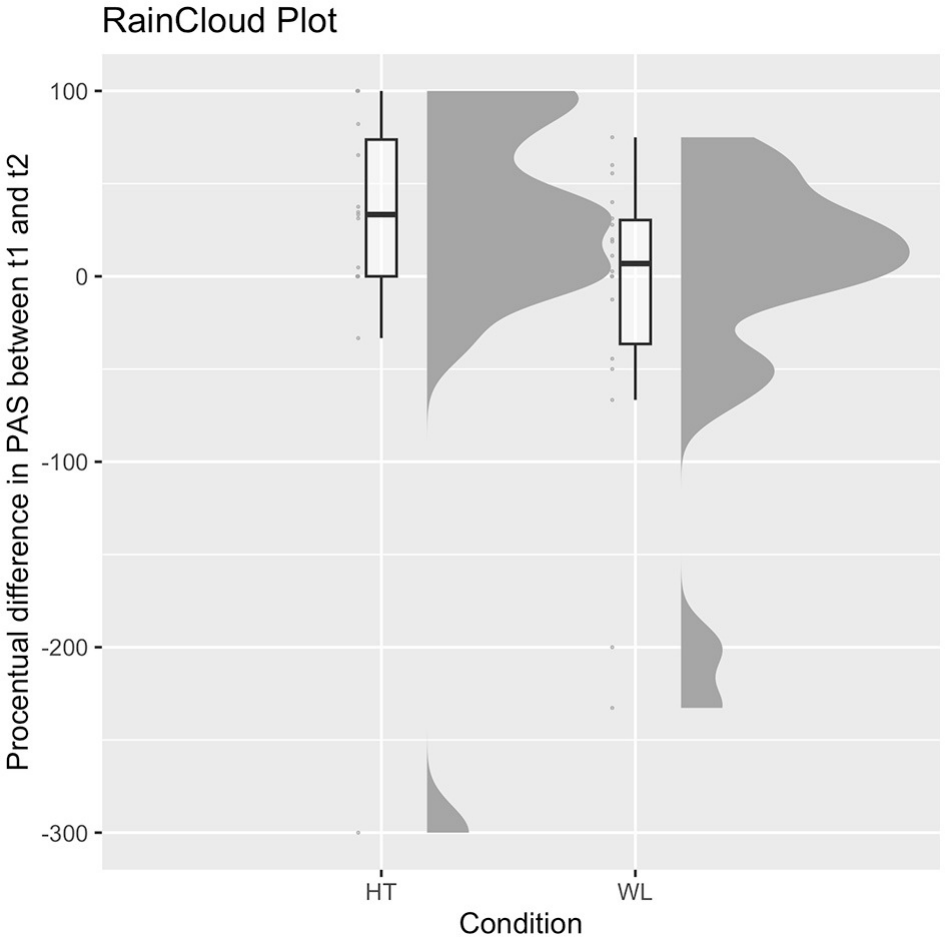
Medians and distributions of both groups in the percentage symptom reduction in PAS in the PP sample and individual scores. HT, hypnotherapy; WL, waitlist control group.

The median percentage symptom reduction in the PAS score between baseline and the end of treatment was between Md = 33.97% (range 400; −300–100) and Md = 36.05% (range 424.47; −300 to 124.47) in HT and Md = 6.90% (range 307.72; −232.72 to 75.00) in WL in the ITT sample. Only one of the five *U*-tests calculated for each imputed dataset separately showed a non-significant difference between the two groups, *U* = 110.50, *p* = 0.052 (one-sided). All others were indicating a higher symptom reduction in the HT compared to the WL group, *U* = 92.50, *p* = 0.014 to *U* = 106.50, *p* = 0.040. A non-significant result was found in the PP sample. HT (Md = 33.33%, range 400; −300 to 100) did not differ from WL (Md = 6.90%, range 307.72; −232.72 to 75.00), U = 90.50, *p* = 0.054 (one-sided). Results of the PP sample regarding medians and distributions of the percentage symptom reduction in both groups as well as individual scores are displayed in Figure 2.

### 3.4. Secondary outcomes

As secondary analyses, we conducted rmANOVAs with the PAS scores of t1 and t2, see Table 2 for the descriptive statistics. In three of the five imputed datasets, we found a significant interaction of time and treatment condition in the ITT sample, *F*_(1, 34)_ = 4.70, *p* = 0.037, partial η2 = 0.12, to *F*_(1, 34)_ = 5.87, *p* = 0.021, partial η^2^ = 0.15, indicating that HT showed a higher symptom decrease compared to WL. In the PP sample, *F*_(1, 31)_ = 3.10, *p* = 0.088, partial η^2^ = 0.09, no significant interaction could be identified. With overall 36 patients (18 per group), a repeated-measures ANOVA with two groups and two measures (pre and post) with an observed correlation of *r* = 0.40 between measures to show an effect of *d* = 0.25 or higher using an alpha of α =0.05 had a power of 79% (1–β) (non-centralized-parameter δ = 8.18, critical *F* = 4.13).

The symptoms of the ITT sample decreased in HT with an effect size of Cohen's *d* = −0.82 and in WL with *d* = −0.11. The effect for the difference in the t2 PAS score between both groups was *d* = 0.49. The further symptom improvement in the HT patients until the 3-month follow-up (t3) had an effect size of *d* = −0.16. In WL patients, after receiving HT, the symptoms decreased with an effect size of *d* = −0.50. The difference between both groups at t3 was *d* = 0.09.

In the PP sample, HT showed a symptom decrease from t1 to t2 with an effect size of Cohen's *d* = −0.73, and WL with an effect size of *d* = −0.11 regarding the imputed data. The difference in the PAS t2 score between both groups had an effect size of *d* = 0.56. The symptoms further decreased by approximately *d* = −0.09 in the HT patients until the 3-month follow-up t3. In WL patients, after receiving the HT treatment, the effect size of the symptom improvement was *d* = −0.51. The difference between both groups at t3 was *d* = 0.07.

### 3.5. Satisfaction

Not all patients returned their responses. Due to the nature of the study design, satisfaction ratings for HT and WL patients are described separately. In each study condition, patients rated their satisfaction after the end of treatment. At the end of the treatment (t2), 10 of the HT patients rated the treatment as effective with an average M = 81.70 (SD = 20.94). They rated their therapist as being highly competent with an average M = 97.60 (SD = 5.15). At the end of the treatment in the WL patients (t3), 14 WL patients rated the treatment comparably effective with M = 79.43 (SD = 22.83). They rated their therapist as being highly competent with M = 91.57 (SD = 14.37).

### 3.6. Safety

An overall rate of four SAEs was reported for three different patients. One patient in WL reported a new medical condition (breast cancer) following two hospitalizations for somatic reasons. Another patient in HT was hospitalized for eye surgery. Those SAEs were not treatment-related. One patient with the HT condition reported some potentially trauma-related new PTSD symptoms after the first sessions that were not mentioned before. This patient was hospitalized, she discontinued the study participation because the agoraphobic symptoms were no longer the focus of treatment.

### 3.7. (Epi-)genetic results

Analyses on symptom reduction including pre and post only were conducted with *n* = 17 and including follow-up with *n* = 10, as PAS scores were available for all participants of the subsample for pre and post, but only for 10 at follow-up.

*COMT* Val^108/158^Met genotyping revealed four participants to be homozygous for the Met (A) allele, eight participants were homozygous for the Val (G) allele, and five participants were heterozygote (A/G) carriers. Mean *COMT* DNAm was 49.7% (SD = 9.1%) at pre, 52.0% (SD = 7.7%) at post, and 47.7% (SD = 8.4%) at follow-up and did not differ significantly between the assessed time points. The subsample's mean PAS score was 12.5 (SD = 7.9) at pre, 8.0 (SD = 7.2) at post, and 4.9 (SD = 5.1) at follow-up and decreased significantly from pre to post [*F*_(1, 16)_ = 5.86, *p* = 0.03]. PAS score comparison of all time points revealed a reductive trend [*F*_(2, 18)_ = 3.11, *p* = 0.069]. *COMT* Val^108/158^Met genotype had a significant main effect on DNAm [*F*_(2, 14)_ = 8.15, *p* = 0.004], as well as on PAS score comparing both, pre and post only [*F*_(1, 14)_ = 5.69, *p* = 0.032], and all three time points [*F*_(2, 7)_ = 7.65, *p* = 0.017]. Moreover, PAS score change was not of predictive value for DNAm change, as no significant regression equation was found.

## 4. Discussion

This was the first pilot RCT to investigate the efficacy of a novel hypnotherapeutic treatment approach for agoraphobia patients. We found greater symptom improvement in patients who received the hypnotherapy compared to those who were allocated to a waiting condition in four of the five imputed datasets for the primary outcome and three of the five imputed datasets using RM-ANOVAs. Furthermore, effect sizes suggest a large effect regarding the symptom improvement of patients receiving HT without waiting time compared to those waiting for a treatment who showed only little improvement. After treatment, the difference between groups showed a small to medium effect size (*d* = 0.49–0.56) indicating the superiority of HT. Contrary to our hypothesis, the superiority of HT compared to WL could not be found in the primary outcome in the PP and one of the ITT datasets. The small to medium effect sizes are in contrast to the results of a meta-analysis, which found large effect sizes when comparing CBT to passive control groups for panic disorders, but only medium effect sizes when compared to placebos (e.g., Mitte, 2005). Our a priori power analysis was based on a high effect size (we used *d* = 0.80). Therefore, we conducted two *post-hoc* power analyses. The first was conducted to determine the power of our study based on the sample size and effect size of our study. The *post-hoc* power analysis for the Mann–Whitney *U*-test revealed that for an effect size of *d* = 0.49, the results in 18 patients per group (total 36) achieved a power of 41.0%. The second was conducted to determine the sample size for a study with the originally planned power (86%) together with the effect size found in our study (*d* = 0.49). This power analysis would have required a sample size of 132 patients. In our study, non-specific factors might have influenced a small improvement in the WL control group, such as the prospect of treatment after 12 weeks, regression to the mean, or contact with the staff collecting the (epi-)genetic samples or with the raters for assessing symptoms before and after waiting time. Moreover, the range of symptom change was quite high in the HT condition pointing to individual differences regarding the outcome of the HT treatment. Most of the previous research (Mitte, 2005; Bandelow et al., 2014; Kaczkurkin and Foa, 2015), though, focused on panic disorder with and without agoraphobia, and, thus, results cannot be directly compared to those of our study. In our pilot study, the focus was on agoraphobic patients using the new DSM-5 classifications as a single diagnostic category and only 33% had current panic disorders and 13% had panic attacks. As outlined by Hoffart et al. (2016), agoraphobia without panic disorder is a distinct diagnostic category different from panic disorders which showed less improvement compared to panic disorder. The positive results of the hypnotherapy used in our pilot study compared to a previous RCT on hypnotherapy (Van Dyck and Spinhoven, 1997) can be explained by the additional use of hypnotic regression, that was, in our case, exposition in sensu comparable to imagery rescripting. The central intervention of the HT treatment, the hypnotic symptom regression technique with the following reframing, should be used in future research. However, it cannot be concluded that those elements are really due to changes in the specific therapeutic factor, such as imaginative exposure, or to non-specific factors, as direct comparisons of hypnotherapy to exposure-based treatments, such as CBT, as well as mediator studies are still missing.

Overall, treatment satisfaction was very high, as were completion rates. Only one patient developed new symptoms after the first treatment sessions. No relevant other serious adverse effects were reported. Thus, HT was effective, feasible, and safe in the treatment of agoraphobia patients. Patients with potential trauma-related disorders should be treated differently. The results add to the literature that hypnotherapy can successfully reduce symptoms of anxiety (Valentine et al., 2019). Even more, it is one of the first RCTs to indicate that manualized HT can be used to treat a specific anxiety disorder, that is, agoraphobia.

The *COMT* Val^108/158^Met polymorphism has been widely associated with susceptibility to mental illness (e.g., Hosák, 2007). In particular, the Val allele has been associated with anxiety disorders in various previous studies (Hamilton et al., 2002; Domschke et al., 2004; Taylor, 2018). Furthermore, it has been shown that the Val^108/158^Met genotype is associated with hypnotizability (Lichtenberg et al., 2000; Szekely et al., 2010; Rominger et al., 2014; Storozheva et al., 2018). Although contradicting in terms of the direction of effect, these studies suggest the efficacy of HT partly depends on a patient's genotype. In our study, we also observe an effect of the Val^108/158^Met genotype on the PAS score, indicating that enhanced HT efficacy in patients with a specific *COMT* genotype could be possible. The Val^108/158^Met polymorphism has in addition been reported to effect *COMT* DNAm (Schreiner et al., 2011; Swift-Scanlan et al., 2014; Thomas et al., 2019), and we were able to replicate this effect in our sample. Thus, independent of HT, Val^108/158^Met genotype differences might be interesting to elucidate further regarding susceptibility to psychiatric disorders and DNAm alterations. Our hypothesis of differential DNAm of the *COMT* gene over the course of HT could neither be confirmed nor did we elucidate an association with symptom reduction. Epigenetic mechanisms have previously been proposed to play a role in therapy efficacy. However, as *COMT* DNAm did neither change during therapy nor was of predictive value for therapy response, our study does not provide evidence for an involvement of *COMT* DNAm in biological mechanisms underlying HT efficacy.

### 4.1. Limitations

Due to considerable difficulties in identifying suitable patients, we were only able to enroll 36 of the planned 48 patients in the available 15 months. Despite the efforts to recruit patients and the extension of the official recruitment period to 15 months (October 2018 to January 2020), which meant an additional 6 months of treatment and follow-up afterward (until July 2020), we did not find the number of patients we had targeted. This was probably due to the very strict inclusion and exclusion criteria, such as focusing on a single anxiety disorder (agoraphobia), and excluding patients who had received psychotherapy in the previous 12 months. Another limiting factor was the COVID-19 lockdown in Germany. Probably due to the small sample size, the primary hypothesis was not clearly supported by the results. We could not perform further subgroup analyses, such as for agoraphobia patients with additional panic attacks or panic disorder, or identifying moderators of symptom improvement. Further analyses regarding response rates as recommended by Loerinc et al. (2015) were not performed. Despite the small sample size, effect sizes indicated small to moderate differential effects as well as a large effect of symptom decrease in HT. Effects sizes were lower than expected for HT compared to previous RCTs investigating CBT. This could be due to the fact that the symptoms measured with the PAS were on average small (a score of 7–17) before treatment so patients were less likely to improve much. Regarding the (epi-)genetic analysis, one has to be aware that the results need to be interpreted with caution, as the sample size was small. We therefore recommend viewing this part of the study as exploratory analyses that could inspire further research in a larger cohort with balanced numbers of participants' genotypes, as well as adequate numbers of male and female participants to determine implicated sex differences. In our sample, the proportion of female patients (80.6%) compared to male patients was much higher than the prevalence rates found in Germany, where female patients had two to three times higher rates than male patients (Jacobi et al., 2014). Thus, the results of our study should not be generalized before confirmation in other RCTS and samples.

## 5. Conclusion

The results can be interpreted as a first indication that HT might be a psychotherapeutic method that expands the number of available therapies in the treatment of agoraphobia. Comparisons with other treatments, especially those with *in vivo* expositions, are still lacking. Future studies should also compare efficacy in agoraphobia patients in a larger sample, also allowing for subgroup analyses for patients with comorbid panic disorder or panic attacks.

## Data availability statement

The raw data supporting the conclusions of this article will be made available by the authors, without undue reservation.

## Ethics statement

The studies involving human participants were reviewed and approved by Ethics Committee of the University Hospital Tuebingen. The patients/participants provided their written informed consent to participate in this study.

## Author contributions

KF and ABa wrote the study protocol. KF wrote the first draft of the manuscript, conducted the statistical analyses of the efficacy study results, and was involved in the data collection of the project. ABe and AW were involved with the analyses of the (epi-)genetic data and the writing of the respective paragraphs. PJ wrote the treatment manual, was involved as a supervisor in the trial, and helped with the description of the treatment. MD helped with the statistical analysis and Figure 2. BC and CS conducted the study treatment (therapists) and gave feedback on the current form of the manuscript. BK was involved in the data collection and gave feedback on the current form of the manuscript. VN was the head of the (epi-)genetic project in a subsample of the study and supervised the conduction of the project, the writing, and analyses of the respective paragraphs. ABa supervised the progress of the study and the current manuscript. All authors contributed to the article and approved the submitted version.
